# Quantification of Caffeine Interactions in Choline Chloride Natural Deep Eutectic Solvents: Solubility Measurements and COSMO-RS-DARE Interpretation

**DOI:** 10.3390/ijms23147832

**Published:** 2022-07-15

**Authors:** Tomasz Jeliński, Piotr Cysewski

**Affiliations:** Department of Physical Chemistry, Pharmacy Faculty, Collegium Medicum of Bydgoszcz, Nicolaus Copernicus University in Toruń, Kurpińskiego 5, 85-950 Bydgoszcz, Poland

**Keywords:** caffeine, solubility, NADES, choline chloride, COSMO-RS, DARE

## Abstract

Solubility of active pharmaceutical ingredients is an important aspect of drug processing and formulation. Although caffeine was a subject of many studies aiming to quantify saturated solutions, many applied solvents suffer from not being environmentally friendly. This work fills this gap by presenting the results of solubility measurements in choline chloride natural deep eutectic solvents, ccNADES, comprising one of seven of the following polyalcohols: glycerol, sorbitol, xylitol, glucose, sucrose, maltose and fructose. The ratio of ccNADES components was optimized for maximizing caffeine solubility at room temperature. Additionally, temperature dependent solubility was measured for the first four systems exhibiting the highest solubility potential, both in their neat forms and in mixtures with water. Results were used for intermolecular interactions assessments using the COSMO-RS-DARE approach, which led to a perfect match between experimental and computed solubility values. An important methodological discussion was provided for an appropriate definition of the systems. Surprising linear trends were observed between the values of fitting parameters and water-ccNADES composition. In addition, comments on selection of the values of the fusion thermodynamic parameters were provided, which led to the conclusion that COSMO-RS-DARE solubility computations can effectively compensate for the inaccuracies of these important physicochemical properties.

## 1. Introduction

Caffeine (1,3,7-trimethylxanthine) is a stimulant of the central nervous system belonging to the class of methylxanthines. This group of compounds is widespread in the natural environment and can be found in tea and other plant leaves, coffee and cocoa beans, as well as cola seeds [1]. Methylxanthines can be classified as purine-based alkaloids, having a fused heterocyclic system in their moiety, which contains a pyrimidine and an imidazole ring [2]. The general properties of methylxanthines include rather poor solubility in water or ethanol [3] and low values of water-octanol partition coefficient [4]. Caffeine can be found in over sixty different plants, which usually contain other methylxanthines as well [5,6]. It has been used as a traditional beverage for thousands of years [7,8] and it is currently a very popular [9,10] physical [11,12] and cognitive [13,14] performance enhancer, although its immoderate consumption is related to some side effects [15,16,17]. Caffeine after oral intake is readily and completely absorbed from the small intestine [18]. It is then metabolized and in the form of mainly paraxanthine excreted with urine [19]. Similarly, as other methylxanthines, it binds with adenosine receptors [16] and induces psychomotor stimulant properties in the brain [18]. Caffeine is also used in different medical applications, namely in combination with painkillers, e.g., to relieve migraine [20,21], in bronchopulmonary dysplasia treatment [22], orthostatic hypotension treatment [23] and the treatment of apnea of prematurity [24].

Limited solubility of active pharmaceutical ingredients (APIs) in different solvents is a well-known difficulty in the pharmaceutical industry [25]. The problem is evident when looking at the Biopharmaceutics Classification System (BCS), according to which even 40% of APIs may be treated as almost insoluble in water [26] and possibly even 70% of drugs under development [27]. Because of this, many different strategies were proposed to address this limitation. This includes amorphization [28,29], formation of monocrystals [30,31], micronization [32] and solid dispersion formation [33], complexation with cyclodextrins [34,35], salts [36,37] and cocrystals [38,39] formation. Another way of improving solubility involves cosolvation techniques in which a particular amount of a solvent is added to the primary solvent [40,41,42] resulting in elevated solubility. Unfortunately, the use of organic solvents in this form may be limited due to their potential toxicity, which requires the search for alternatives.

According to the concept of green chemistry [43,44], in order to minimize the negative influence of the chemical industry on the environment, there is a need to reduce or even eliminate the usage of hazardous chemical substances including solvents, which should be replaced with environmentally friendly green solvents [45,46]. Apart from ionic liquids (IL) and supercritical fluids (SCF), the concept of green solvents also includes deep eutectic solvents (DES), which are mixtures of two or more individual components with a melting point of the mixture lower than the ones of its components [47,48]. While sharing many features with ionic liquids, DES are distinguishable from them mainly by the fact that they usually include non-ionic species as their constituents [49]. Natural deep eutectic solvents (NADES) can be regarded as a sub-type of DES and differ from other eutectic solvents by the fact that they include organic acids, alcohols, amino acids, sugars or other plant-based primary metabolites as their components [50,51]. A very popular compound used in NADES is choline chloride, the advantages of which include its non-toxicity and low cost [52]. NADES experience many beneficial properties which include low volatility, sustainability and biodegradability, low cost and simplicity of preparation, as well as the potential of fine-tuning for specific applications [53,54,55,56]. These beneficial properties make NADES useful in a variety of applications, including the pharmaceutical industry [57,58,59,60,61].

The solubility of caffeine was studied experimentally in different systems, including neat organic solvents [62,63,64], binary solvent mixtures [65,66,67,68,69,70,71,72,73,74] and carbon dioxide [75,76]. Our research team has recently studied the solubility of caffeine in several aprotic proton acceptor solvents and their mixtures with water [77], also offering a tested screening procedure for finding greener solvents. In recent years, due to the increasing demand for alternative solvents, the solubility of caffeine in ionic liquids and NADES also became a subject of interest. These studies were focused mainly on extraction of caffeine from natural sources and involved such media as cholinium-based ionic liquids [78], imidazolium-based ionic liquids [79], amino acid-based ionic liquids [80], systems containing sorbitol [81] and DES [82].

In this study, the solubility of caffeine was measured in several choline chloride-based natural deep eutectic solvents (ccNADES) together with its solubility in ccNADES—water mixtures. The experimental results were interpreted using the COSMO-RS-DARE framework in order to gain insight into the interactions responsible for the actual caffeine solubility in the studied systems. Some important considerations regarding the methodology and accuracy of solubility predictions were also provided.

## 2. Results and Discussion

### 2.1. Solid State Characteristics

According to different studies, caffeine can adopt two stable polymorphic forms and exist as hydrate [83,84,85,86,87], with a third polymorphic form obtained through sublimation [88]. Therefore, the DSC and FTIR measurements were conducted in order to determine which form of caffeine is present in the solid residues left after solubility experiments.

**Figure 1:**
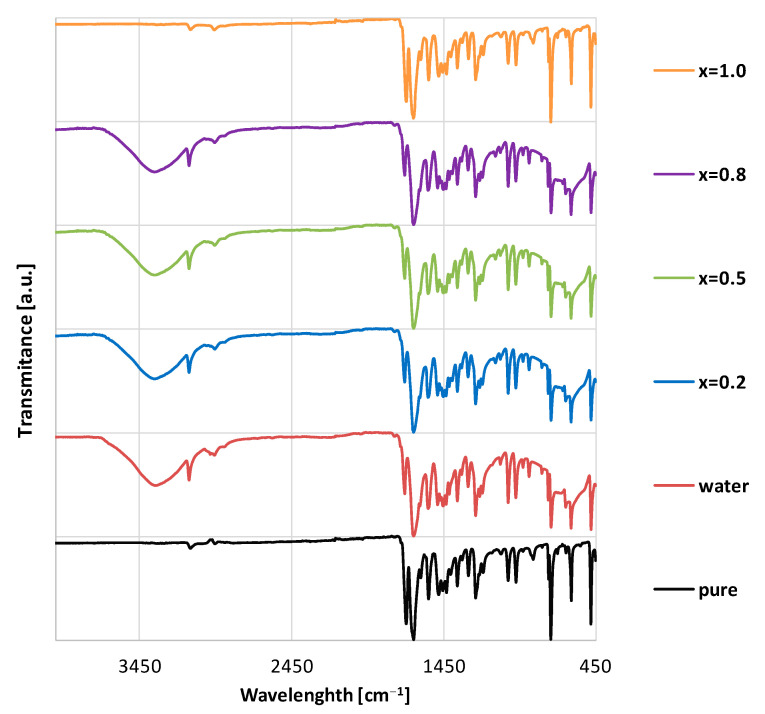
The FTIR spectra of caffeine precipitates after solubility measurements in NADES (choline chloride + glycerol), water and their mixtures. Values in parentheses indicate the mole fractions of ccNADES in solute-free solutions. For comparison, the spectrum of pure caffeine is presented.

FTIR studies have confirmed that in neat solvents caffeine exists as its anhydrous form, while a hydrate is obtained after the addition of water [77,83,84,85]. This is shown with the example of caffeine dissolved in neat NADES comprising choline chloride and glycerol compared to its mixtures with water, as shown on the IR spectra in Figure 1. In the case of pure caffeine powder and the solid residues found after dissolving caffeine in water-free choline chloride-based NADES (x_ccNADES_ = 1.0) the spectra are in good agreement with each other. Several characteristic absorption bands can be attributed to this form of caffeine, namely C-H (alkyl) at around 2900 cm^−1^, C=O at around 1650 and 1710 cm^−1^, C=C at around 1550 cm^−1^ and C-N at around at around 1250 cm^−1^. However, the dissolution of caffeine in water and in mixtures containing both water and ccNADES (x_ccNADES_ = 0.2 to x_ccNADES_ = 0.8) results in the appearance of a new band which is quite wide and has a maximum at around 3400 cm^−1^, which can be assigned as the O-H stretching present in the hydrate [89]. All of the studied ccNADES systems show an analogous behavior as the choline chloride—glycerol systems.

**Figure 2:**
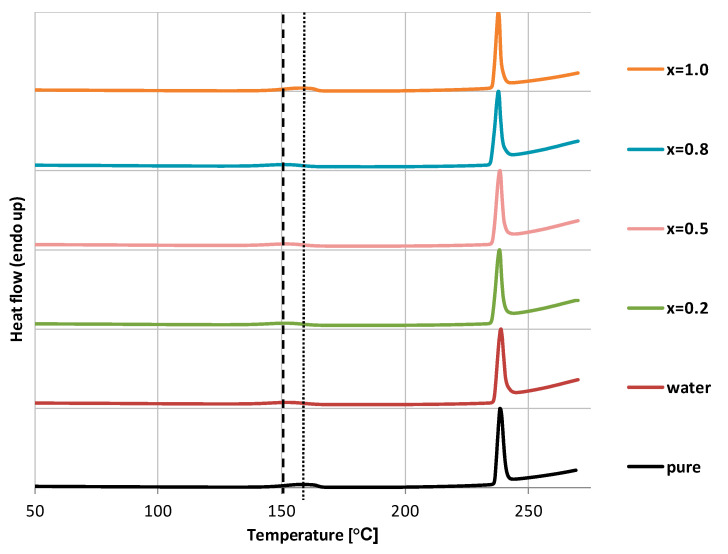
DSC curves of caffeine precipitates after solubility measurements in glycerol containing ccNADES, water and their mixtures. Values in parentheses indicate the mole fractions of ccNADES in solute-free solutions. Vertical lines were drawn for guiding the eye, which correspond to the water loss of the hydrate (dashed line) and the transition between caffeine polymorphic forms II and I (dotted line). The DSC curve of pure caffeine is also provided for comparison.

The results of the DSC studies were consistent with the ones obtained through FTIR measurements. For clarity, only the results obtained for glycerol containing ccNADES and its water mixtures were presented in Figure 2. Both pure caffeine and the precipitates can be characterized by a melting point at circa 238 °C which is in satisfactory agreement with literature data for caffeine polymorphic form labeled as I. The mean value for the whole dataset is 238.24 °C with a standard deviation of 0.42 °C. When taking into account the position of the first endothermic signal there are however significant differences among the studied samples. Pure caffeine and the samples obtained after dissolution in neat ccNADES are characterized by a peak with a mean value of 157.98 °C and a standard deviation of 0.46 °C. This can be assigned as the transition from the polymorphic form II of caffeine to its form I, however compared to literature this peak is shifted toward higher temperatures. In the case of caffeine solubilized in mixtures containing both ccNADES and water the peak has a maximum located at a mean value of 151.58 °C with a standard deviation equal 0.59 °C, which once more is somewhat shifted compared to literature data. Nevertheless, it can be attributed to the loss of water experienced by the caffeine hydrate. It seems therefore that both FTIR and DSC measurements give a consistent picture, pointing out the fact that the presence of water in the mixtures is responsible for the formation of a hydrate, while the polymorphic form II of caffeine is the result of crystallization in the case of absence of water in the systems.

### 2.2. Experimental Solubility of Caffeine

As it was said before, caffeine was the subject of numerous solubility studies in neat and mixed solvents and authors also contributed to this field [77] with already published results which confirm the validity of the approach applied here. This was established by comparing the obtained solubility values and the ones provided in literature [62,63,64] with Table S1 in Supplementary Materials showing detailed results of this comparison. Since there are no reported data regarding caffeine solubility in the ccNADES systems studied here, measurements in water and some organic solvents were used. Our protocol was also successfully applied for the characteristics of other compounds dissolved in ccNADES including theophylline [90], theobromine [91], curcumin [92] and sulfonamides [93], which further confirms the adequacy of the used methodology.

**Figure 3:**
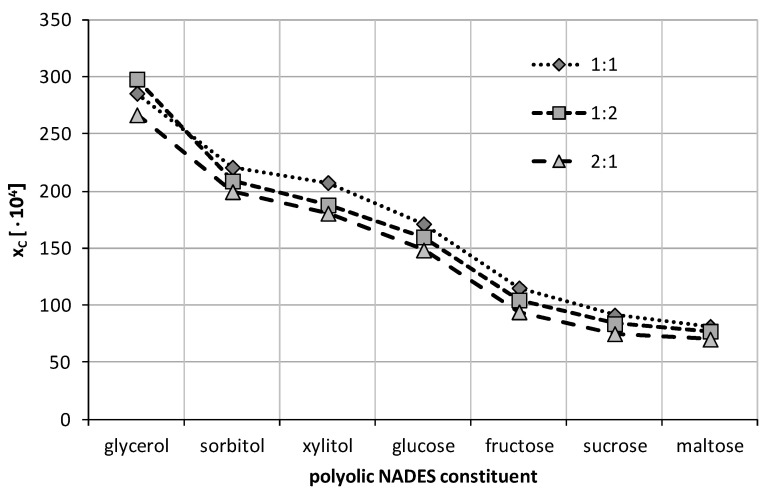
Mole fraction solubility of caffeine at 25 °C determined in water-free ccNADES prepared in varying molar proportions of choline chloride to sugar.

Although it is expected that in general the solubility of many organic solutes in ccNADES is higher compared to traditional organic solvents and their mixtures [90,91,92,93], the selection of the optimal composition is not a straightforward task. Hence, in the first stage, the experimental pre-screening was performed using binary solvents comprising choline chloride and one of the seven studied polyols in three different molar ratios. The results of this preliminary testing performed at 25 °C were collected in Figure 3. Three different molar ratios were tested, namely a unimolar proportion of both constituents, as well as 2:1 excess amounts of either of them.

From the obtained data it can be inferred that in most cases the 1:1 molar ratio of choline chloride and the polyolic constituent yields the highest solubility of caffeine. However, for the ccNADES system including glycerol the composition with an excess amount of the polyol is responsible for the most efficient caffeine solubilization. In this case, the mole fraction of caffeine in the solvent was found to be x_C_ = (297.97 ± 0.70)·10^−4^, which stands for almost a 15-times increase in solubility compared to water. All the other solvents were most efficient in their unimolar composition. The following concentrations were found for saturated systems of caffeine in ccNADES comprising sorbitol (x_C_ = (220.77 ± 0.23)·10^−4^), xylitol (x_C_ = (207.36 ± 0.82)·10^−4^) and glucose (x_C_ = (171.22 ± 0.58)·10^−4^). This yields an increase of solubility compared to water from 8.5- to 11-times. Detailed values of the mole fractions of caffeine in the above solvents together with their standard deviations can be found in Table S2 in Supplementary Materials.

**Figure 4:**
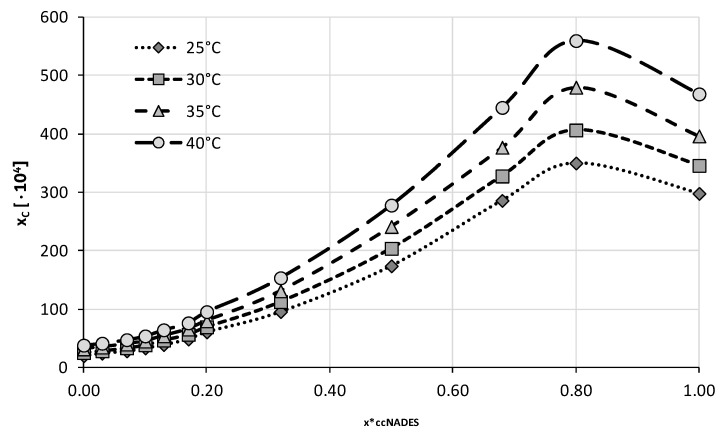
Mole fraction solubility of caffeine in mixtures of water and ccNADES comprising choline chloride and glycerol in 1:2 molar ratio. On the abscissa, x*_ccNADES_ represents the mole fractions of the ccNADES in solute free binary solutions.

**Figure 5:**
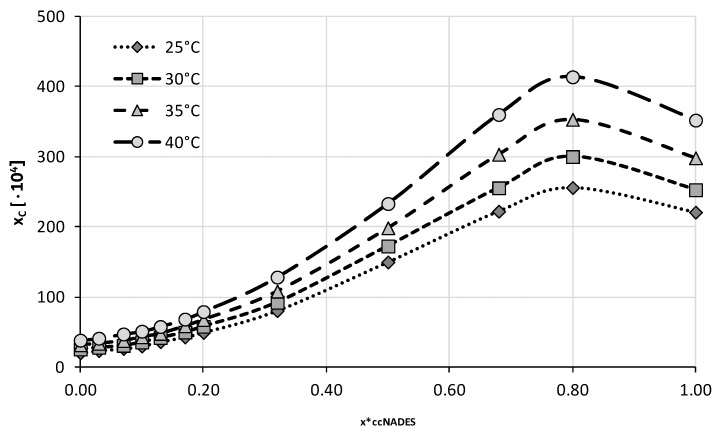
Mole fraction solubility of caffeine in mixtures of water and NADES comprising choline chloride and sorbitol in 1:1 molar ratio. On the abscissa, x*_ccNADES_ represents the mole fractions of the ccNADES in solute free binary solutions.

**Figure 6:**
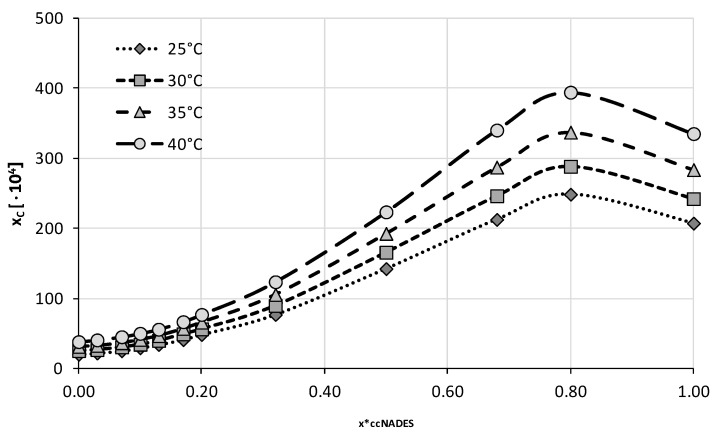
Mole fraction solubility of caffeine in mixtures of water and ccNADES comprising choline chloride and xylitol in 1:1 molar ratio. On the abscissa, x*_ccNADES_ represents the mole fractions of the ccNADES in solute free binary solutions.

**Figure 7:**
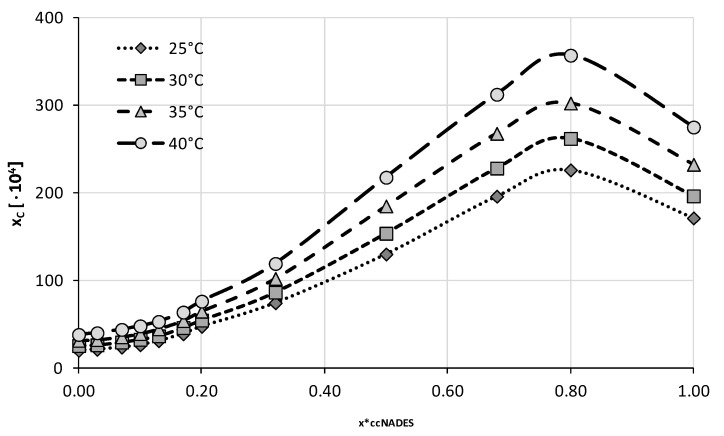
Mole fraction solubility of caffeine in mixtures of water and ccNADES comprising choline chloride and glucose in 1:1 molar ratio. On the abscissa, x*_ccNADES_ represents the mole fractions of the ccNADES in solute free binary solutions.

The four optimal compositions were chosen for the next stage of experiments in which the ccNADES were treated as cosolvents and mixed with water in varying proportions. A total of twelve different compositions were studied including both neat ccNADES and water. Four different temperatures from 25 °C to 40 °C with 5 °C intervals were studied in this series of measurements. The obtained results were collected in Figure 4, Figure 5, Figure 6 and Figure 7 and the corresponding detailed values of caffeine mole fractions can be found in Supplementary Materials, Tables S3–S6.

When taking into account the obtained solubility profiles, a similar picture emerges for all four studied ccNADES systems. It turned out that a relatively small amount of water added to the NADES promotes the increase of caffeine solubility. For all of the studied systems, the highest solubility was obtained when the amount of ccNADES in the solution was equal x*_ccNADES_ = 0.8. The order of increased solubility of caffeine found for pure ccNADES remains the same in their mixtures with water and the increase in temperature was responsible for elevated solubility of caffeine. As mentioned beforehand, the NADES based on choline chloride and glycerol resulted in the highest solubility of caffeine, i.e., x_C_ = (559.49 ± 2.06)·10^−4^ at 40 °C, which was 19% higher than the pure ccNADES. For sorbitol and glycerol, the most effective compositions responsible for caffeine solubility were equal to x_C_ = (413.65 ± 2.09)·10^−4^ and x_C_ = (394.23 ± 2.43)·10^−4^ at 40 °C, respectively. The least effective composition involving glucose as the second constituent yielded caffeine solubility equal to x_C_ = (356.94 ± 1.17)·10^−4^ also at 40 °C, which amounted to an almost 30% increase in solubility compared to pure ccNADES. While the solubility increase in caffeine in these mixtures is not very pronounced, it is worth noting that an addition of small amounts of water to the system is a factor improving the handling of the systems due to decreased viscosity and density. Moreover, problems with high hygroscopicity of ccNADES systems become omitted by such optimal formulations.

When comparing the results obtained in this study with solubility of caffeine in organic solvents [77], it is evident that ccNADES perform generally better, both in their neat form and when considering mixtures with water. For example, at 25 °C the solubility of caffeine in NADES containing choline chloride with glycerol (the most effective among studied neat ccNADES) is 165% of caffeine solubility in pure DMSO, which was found to be the best performing organic solvent. Among the organic solvents only DMSO and DMF can compete in solubilization efficiency with the studied ccNADES. Since both of these solvents are related to serious toxicity issues [94,95], the choice of NADES as solubilizing media is even more evident.

### 2.3. Intermolecular Interactions of Caffeine with Solvents Components

**Figure 8:**
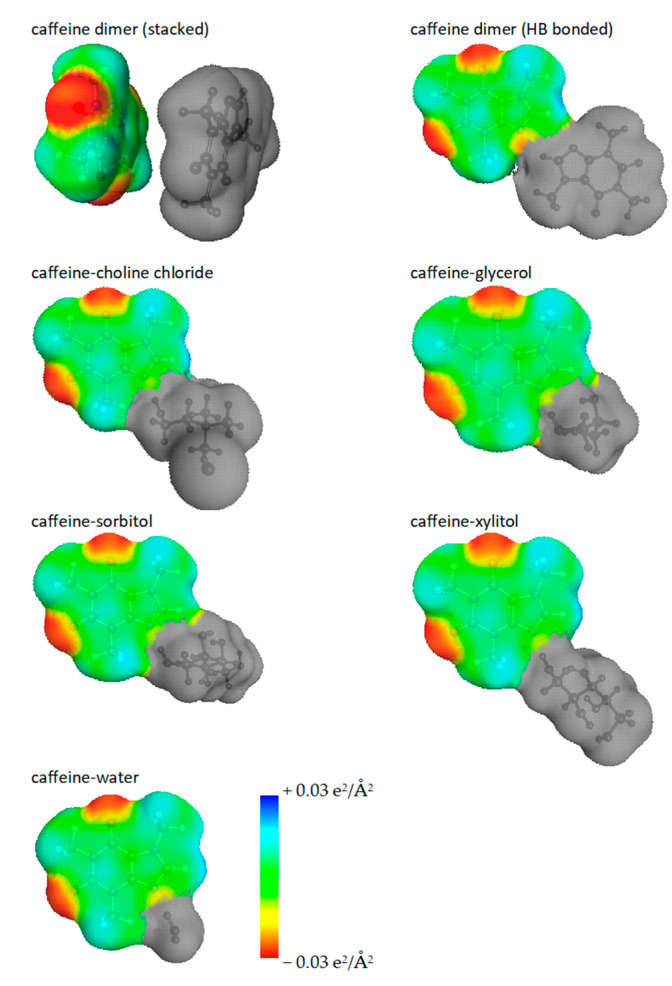
The graphical representation of direct caffeine contacts in ccNADES in the form of “*mcos*” files used for extension of the pool of caffeine conformers in COSMO-RS-DARE computations. The gray part corresponds to forcing the zero-charge setting on all atoms not belonging to caffeine for preserving the formal total charge and atom numbers as monomeric caffeine.

The observed high solubilizing nature of studied ccNADES indicates that caffeine can strongly interact with solvent constituents. These interactions can be quantified by inspection of the structure and energetics of pairs identified via conformational analysis. Among many potential intermolecular complexes, the most energetically favorable ones are shown in Figure 8. The electron density distributions are presented in the form of so-called “*metafiles*” representing caffeine conformers involved in the intermolecular complex formation in ccNADES solutions. These caffeine conformers are not considered during typical conformational analysis and are added as a result of clusters examination. Hence, the optimization of pairs leads to eventually new geometries with alternative electron density distributions compared to monomeric caffeine. The way of presentation offered in Figure 8 emphasizes that interactions of caffeine with solvent molecules extend the pool of potential diversity of solute structures in the solutions. It is interesting to note that the most favorable caffeine dimer adopts a stacking conformation. In addition, the alternative hydrogen bonded pair has been found but it happens to be less stable by 1.6 kcal/mol. However, all the most stable pairs formed by caffeine with ccNADES constituents are stabilized by a strong hydrogen bond formed between the N-acceptor center of caffeine located on imidazole ring and the hydrogen atom of hydroxyl group of solvent molecule. The values of the most important energetic contributions to the stabilization of caffeine—ccNADES pairs are collected in Table 1. The last column comprises the final affinity values including all corrections, as enumerated in the methodology section. The most obvious conclusion drawn from the presented data is that polyalcohols have the highest affinity toward caffeine while the interactions of caffeine with choline chloride or water are much less attractive. Dimerization of caffeine is characterized by the lowest affinity among studied intermolecular complexes. From the fact of affinity discrepancies arises a methodological question of the number of structures to be included for adequate representation of caffeine diversity in the pool of conformers. It is important to emphasize that the origin of COSMO-RS–DARE [96] is the observation that interactions occurring in the solutions and leading to new species can alter electron density distributions due to new compound formation. These new species are not included in the monomer pool of conformations resulting from default conformational analysis which might result in misrepresentation of the chemical diversity of the analyzed compound. Hence, a detailed analysis of the consequences of caffeine interactions in ccNADES on corresponding σ–profiles should be performed prior to COSMO-RS-DARE applications.

**Figure 9:**
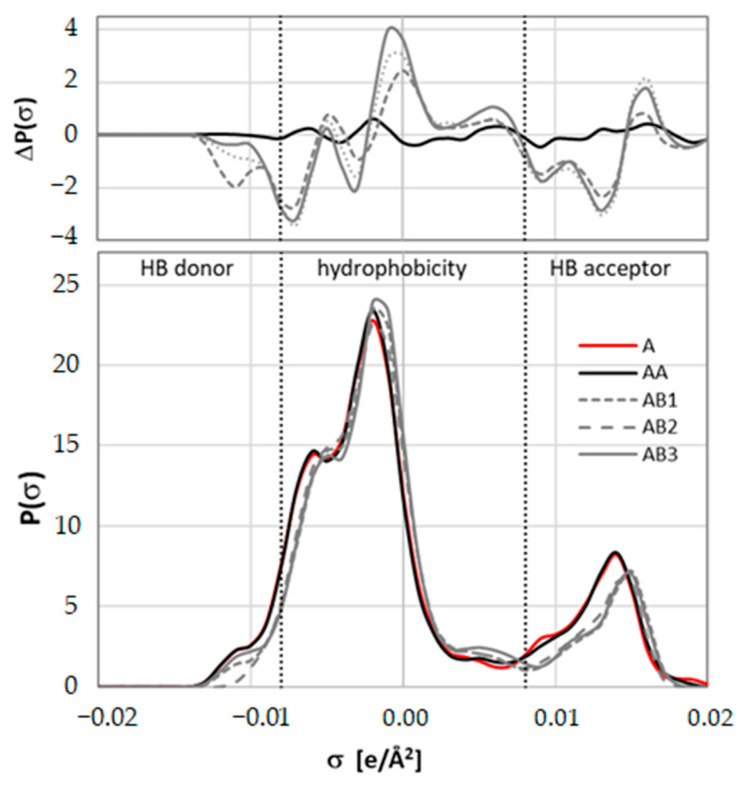
The σ-profiles characteristics of caffeine (A), caffeine dimer (AA), caffeine-choline chloride (AB1), caffeine-glycerol (AB2) and caffeine-water (AB3). Bottom panel represents electron charge density profiles obtained using “*mcos*” files for all pairs graphically presented in Figure 8. Top panel presents relative profiles with respect of monomeric caffeine.

**Figure 10:**
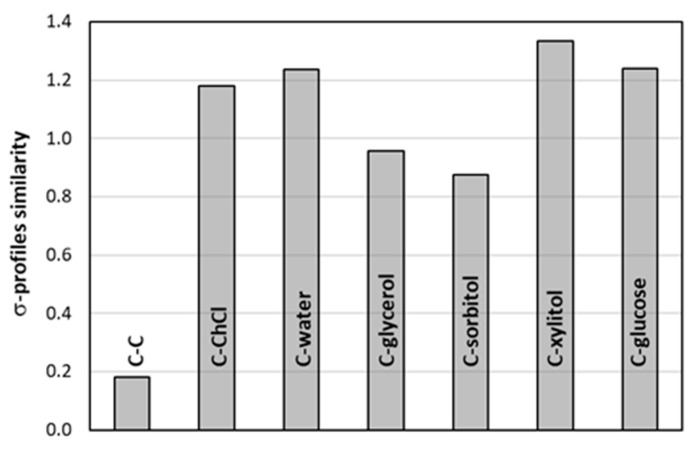
Comparison of σ-profiles similarities of studied complexes of caffeine with ccNADES constituents and water. As a measure of similarity, the RMSD (root-mean-square deviations) values were computed for σ-profiles presented in Figure 9 of pairs (“*mcos*” file) with respect to monomeric caffeine (“*cosmo*” file).

Hence, the σ-profiles were generated for all pairs and compared to the one obtained for monomeric caffeine. The results are presented in Figure 9 and Figure 10. The former exemplifies detailed σ-profiles plots for glycerol containing system. It is quite visible that dimerization is the only case for which no significant changes in σ-profiles are observed. This is particularly evident on the top panel of Figure 9 presenting differential σ-profiles obtained after subtracting one of the caffeine monomers from the one obtained for pairs. For the rest of complexes, a non-trivial alteration of charge distributions takes place. The dotted lines split the region of σ-profiles into three sections typically associated with hydrogen donating and accepting capabilities separated by a hydrophobicity region. It is worth noting that all three regions are strongly affected upon pair formation which suggests that such conformers should not be removed from solubility computations. The changes of HB accepting capability of caffeine after binding to ccNADES is quite expected due to modification of densities on the imidazole ring after binding with the hydroxyl group of ccNADES constituents. Less obvious are however, the other alterations. Negative values on differential plots of σ-plots indicate decreasing electron density of caffeine involved in pairs formation compared to monomeric caffeine. First, the hydrogen atom connected to the carbon of the imidazole ring is apparently quite acidic and can be directly involved in hydrogen bonding [77]. This is supposed to be typical for all methylxanthines, as it was already documented for theophylline [90] and theobromine [91]. Hence, the pair formation with proton donors also reduces the donating character of this acidic center. A bit surprising is the increase in hydrophobicity of caffeine bound to ccNADES constituents or water, which can be attributed to the overall withdrawing of the electron density from the rest of the caffeine molecule not directly involved in the intermolecular complex formation. On the other hand, caffeine dimerization introduced no significant change in the charge distribution, so adding it to the overall pool of conformers is supposed to be superfluous. On the contrary, the pairs of caffeine with water or ccNADES constituents should be included in COSMO-RS DARE computations as introducing new density distribution characteristics of caffeine. However, these σ-profiles, although different from monomeric caffeine, are quite similar to each other, as documented by RMSD values presented in Figure 10. This suggests the possibility of serious simplifications of COSMO-RS DARE computations. In principle, each new conformer resulting from new complex formation can be characterized by introducing a new set of interaction parameters, as defined in Equation (1). However, due to similarities of σ-profiles associated with pairs formation, in all further computations only one set of adjustable parameters was used for the representation of intermolecular interactions with new caffeine conformations. It happened that this simplification does not reduce the accuracy of the computed values of solubility and provides averaged interaction parameter for a given caffeine-ccNADES-water system. The above section provided important methodological clues for efficient COSMO-RS-DARE computations. To complete this part, the exemplary input files were included and described in the Supplementary Materials for ensuring the reproducibility of the results presented here (see Scheme S1 and Table S7).

### 2.4. Results of COSMO-DARE Computations

**Figure 11:**
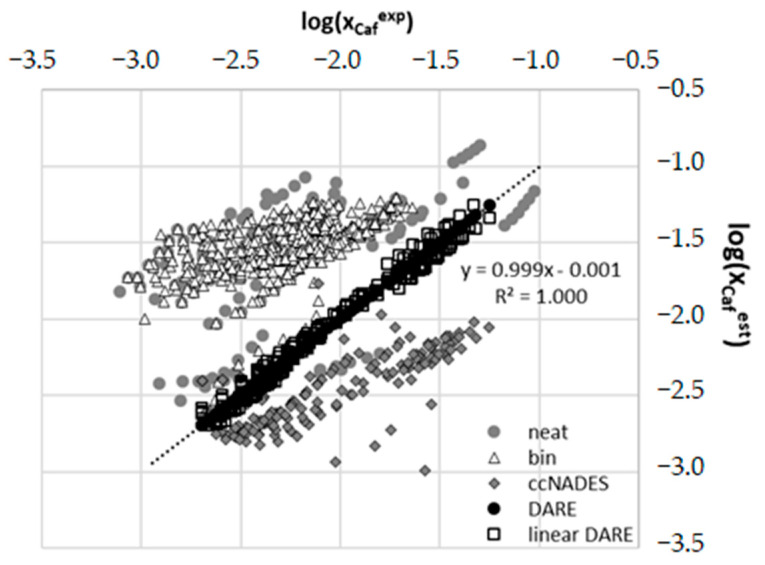
The comparison of accuracy of COSMO-RS computed solubilities of caffeine in neat and binary solutions with experimental data. Additionally, two COSMO-RS-DARE extensions were included, namely first one resulting from the full optimization and the second one corresponding to *G^int^* expressed as split linear functions with values documented in Table 2.

Successful solubility computations within the COSMO-RS framework require additional characteristics of solid species. It is known [97,98] that although the COSMO-RS approach is formulated for liquid bulk systems, solids can also be treated if missing thermodynamic data are provided from external sources [99]. According to details provided in the methodology section, the solubility computations are performed as an iterative procedure seeking convergence of the computed values of chemical potentials until convergence is achieved, as defined in Equation (4). Unfortunately, such default computations are often far from accurate, although for some systems one can observe a fairly quantitative correspondence. For example, solubility of benzamide and salicylamide [100] can be quite accurately predicted without additional parametrization in neat and binary solvents. However, estimations made for a very similar compound, ethenzamide, are much less accurate [101]. It was already documented that the DARE extension of COSMO-RS computations can be quite effective, as it was in the case of nicotinamide, ethenzamide, sulfonamides and methylxanthines [91,101,102,103]. Unfortunately, for the majority of cases, default COSMO-RS is able to predict solubility at most qualitatively. This is also the case for caffeine. In Figure 11, the results of solubility computations of this solute for series of neat and binary solvents are presented and compared to available experimental data. The list of considered here systems is provided in the Supplementary Materials (see Tables S8 and S9). Comparison of computed and experimental data taken from the literature definitively exposes the inadequacy of the COSMO-RS model to describe the solubility of caffeine in neither neat nor binary solvents. In addition, the solubility values computed for caffeine in ccNADES both via traditional COSMO-RS and with COSMO-RS DARE are also provided in Figure 11.

**Figure 12:**
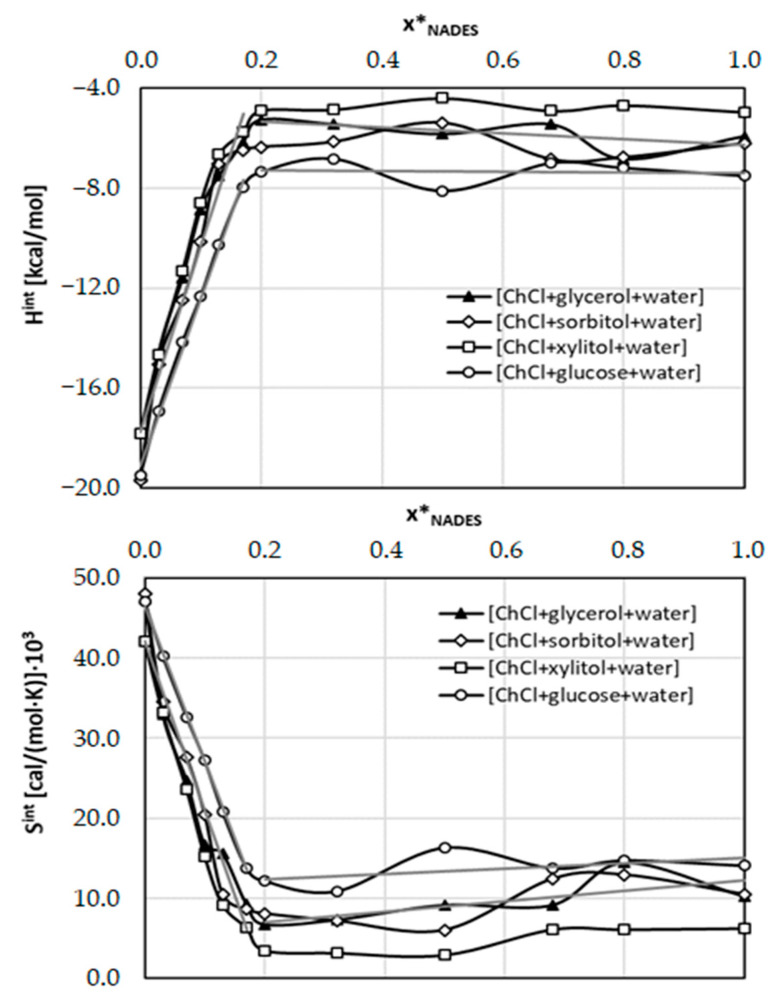
The optimal values of adjustable intermolecular parameters *H^int^* and *S^int^* introduced in the COSMO-RS-DARE approach for caffeine solubility computations in ternary systems comprising water.

A perfect match between experimental values and COSMO-RS-DARE computed solubility of caffeine in ccNADES is clearly visible. On the contrary, the results obtained for neat and binary solvents using the default COSMO-RS approach are rather non-acceptable. Errors are very high and even qualitative solubility analysis seems to be dubious. From this perspective, the advantage of DARE extension is unquestionable. The only problem with these kinds of computations is the necessity of using two fitting parameters for every solution. Indeed, the observed significant improvement in the case of COSMO-RS DARE is granted from optimizing values of intermolecular interaction parameters, *G^int^* = *H^int^* + *T*·*S^int^*. In principle, variations of ccNADES + water composition alters the fundamental physicochemical properties of the resulting ternary solvents and each one is to be treated as a separate solvent system. Hence, for every solute-solvent system there are introduced two fitting parameters including the correction for the energetics of intermolecular interactions of those conformers which were added to the model as a consequence of intermolecular complex formation in the solutions. The values of these parameters were optimized by fitting to experimental data. Resulting optimized values are collected in Figure 12. It is interesting to see that in pure water the values of adjustable parameters adopt the lowest values and systematically and nearly linearly increase with amounts of added ccNADES up to the molar fraction equal to 0.2. Then an almost constant trend is noticed irrespectively of the amount of water. Observed trends can be quantified by linear function of the NADES composition. This was illustrated by gray lines in the case of a system comprising glycerol or glucose. The other two systems behave similarly. Additionally, parameters characterizing observed linear functions are collected in Table 2. The accuracy of such a simplified model was tested by additional computations using *G^int^* values computed from data provided in Table 2. The result of the simplified COSMO-RS-DARE were provided in Figure 11. It is interesting to notice that the loss of accuracy is not very high and correspondence with experimental data is acceptable.

The splitting of the linear trend for x^*^_ccNADES_ around 0.2 can be explained by comparison of intermolecular stabilization of caffeine complexes with water and polyalcohols. As it was documented in Table 1, the caffeine affinity to any sugar predominates in all systems. That is why in the case of sufficient concentration of polyalcohol in the system these kinds of contacts determine the *G^int^* values. On the other hand, increased dilution with water reducing the concentration of sugars systematically diminishes their stabilizing role in ccNADES. This observation is very useful since it enables a significant reduction of the number of adjustable parameters with quite small loss of accuracy.

### 2.5. Role of Fusion Data in Solubility Model

In final comments the influence of accuracy of fusion data on solubility computations is discussed. It was already mentioned that fusion properties, including melting temperature, fusion enthalpy or change of heat capacities upon melting, are added to the COSMO-RS model as external parameters for determining the values of Gibbs free energy, *ΔG_fus_*. This value is used for definition of the reference state for computed chemical potentials of the solute in the bulk phase of a given composition. From general thermodynamics consideration [104], the chemical equilibrium is obtained in the case of equal values of chemical potentials of the pure solid solute and dissolved state. There is a long discussion in the literature [105,106] about the proper way of computation of this important thermodynamic quantity. The problem is additionally complicated by the fact that in many cases fusion data are not directly measurable due to solid sublimations [107], thermal instabilities, polymorphism and formation of a variety of solvates, as for example curcumin [108], fullerene C60 [109], nitrofuran antibiotics [110], L-norvaline [111], β-Alanine [112] among many other cases.

Lack of fusion data is a direct obstacle in using COSMO-RS and available predictive QSPR [99] or additive [113,114] models are quite inaccurate and do not help resolving the problem. Even in the case of known experimental data characterizing fusion properties, their variety obtained by different authors often results in differing values. The most spectacular example is benzoic acid, for which one can find tens of different values of heat of fusion and melting temperatures [115]. Since solubility computations in the COSMO-RS approach are very sensitive to the values of *ΔG_fus_*, the knowledge of these values is mandatory. This problem is also important for COSMO-RS-DARE computations. In this work the values of fusion data were assumed as a mean value of published data, which are collected in Table 3.

**Figure 13:**
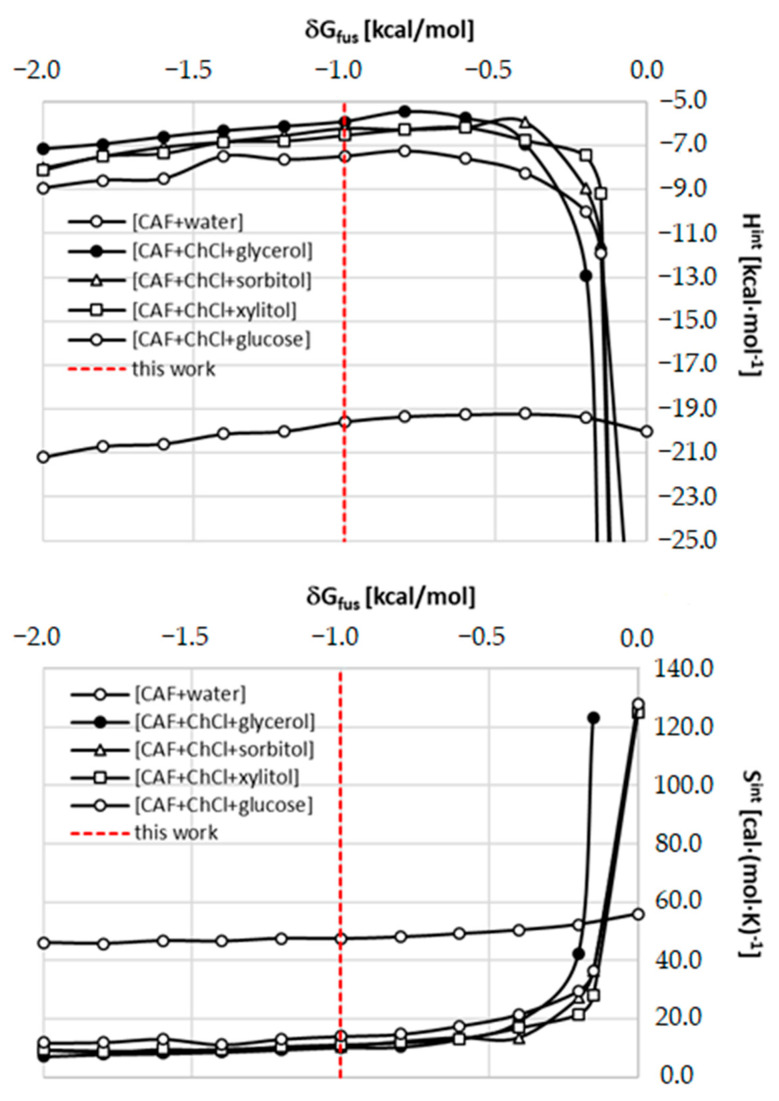
The correlation between the COSMO-RS DARE interaction parameters and fusion data Δ*G_fus_* = Δ*G°_fus_* + δ*G_fus_*, used for solubility computations. The value of Δ*G°_fus_* corresponds to experimental fusion data provided in Table 3.

It is interesting to quantify the sensitivity of *G^int^* to the values of *ΔG_fus_*. For this purpose, COSMO-RS-DARE was used with different values of Gibbs free energies for the most representative binary systems. The obtained results are provided in Figure 13. From these results very interesting conclusions can be drawn. Although in general the values of intermolecular parameters are sensitive to fusion data, there are quite large ranges of shifting values, *δG_fus_*, for which almost identical results are obtained. This is a very important conclusion suggesting a direct applicability of COSMO-RS-DARE to systems for which fusion data are not available or inaccurate. It is then possible to compute solubility for such solids for which either precise fusion data are missing or there are available data with large discrepancies by rational guesses of melting temperature and fusion enthalpy. Even estimates coming from predictive models can be used as such guess values. For the generalization of intermolecular interaction parameters resulting from the COSMO-RS-DARE approach, it is necessary to add a relationship of the kind provided in Figure 13. The rational guess might be understood as both reasonable values and those belonging to the region of relatively low impact on intermolecular interaction parameters. As it was documented in Figure 13, for caffeine the range of such a rational guess is quite large. It happened that reducing the values of *ΔG°_fus_* by at least of 0.2 kcal/mol is a reasonable presumption for caffeine fusion in the studied systems. It seems to be very interesting to see if this is a general conclusion, which immediately provokes forthcoming studies.

## 3. Materials and Methods

### 3.1. Materials

Caffeine (CAS: 58-08-2, MW = 194.19 g/mol) purchased from Sigma Aldrich (Saint Louis, MO, USA) was the key compound used during the studies. The purity of this chemical was ≥99% according to the supplier.

NADES constituents were also provided by Sigma Aldrich and included: choline chloride 117 (CAS: 67-48-1), glucose (CAS: 50-99-7), fructose (CAS: 57-48-7), sorbitol (CAS: 50-70-4), 118 xylitol (CAS: 87-99-0), maltose (CAS: 69-79-5), saccharose (CAS: 57-50-1) and glycerol (CAS: 119 56-81-5). Methanol (CAS: 67-56-1) was purchased from Avantor Performance Materials (Gliwice, Poland). All of the above compounds had a ≥99% purity and were used without initial procedures, apart from choline chloride which was dried before use.

### 3.2. Preparation of the Calibration Curve

The calibration curve used for the determination of caffeine solubility was the same as in the previous study [77]. The preparation of the curve involved obtaining a stock solution in methanol, successful dilutions and measuring the absorbance values at 272 nm wavelength. Three different curves were prepared in this manner and averaged. Detailed values of absorbance and concentrations are provided in the Supplementary Materials in Table S10, while such statistical parameters as the determination coefficient R^2^, limit of detection (LOD) and limit of quantification (LOQ) of the calibration curve are shown in Table S11.

### 3.3. Preparation of Samples in NADES and Their Mixtures with Water

Choline chloride was the main compound involved in NADES preparation. The second NADES constituent was one of the seven polyols described in the materials section. Choline chloride was mixed in different molar ratios with the second constituent in sealed test tubes and placed in a 90 °C water bath until a uniform solution was formed. The resulting ccNADES were used in their neat form or in mixtures with water to which they were added in different ratios.

An excess amount of caffeine was then added to test tubes containing either the neat ccNADES or the ccNADES-water mixture so that a saturated solution could be formed. The samples were then incubated for 24 h at different temperatures in an Orbital Shaker Incubator ES-20/60 from Biosan (Riga, Latvia). The temperature was adjusted with a 0.1 °C accuracy and varied by no more than ±0.5 °C during the daily cycle. The samples were simultaneously mixed at 60 rev/min during heating. Because of the increased viscosity and density of ccNADES, the samples were centrifuged at 1000 rev/min for 5 min with the use of an EBA 20 centrifuge from Hettich (Tuttlingen, Germany) so that the undissolved precipitate would remain on the bottom of the test tube. Next, the samples were filtered using a PTFE syringe filer with 0.22 µm pore size. The test tubes, syringes, filters, etc. were initially heated at a temperature corresponding to the one of the samples. Finally, a defined amount of the obtained filtrate was transferred to test tubes containing methanol and samples diluted in such way were measured spectrophotometrically. Furthermore, the density of the samples was measured for the determination of the caffeine mole fractions.

### 3.4. Solubility Measurements

The determination of caffeine concentration in the samples was conducted using a spectrophotometric method. The A360 spectrophotometer from AOE Instruments (Shanghai, China) was employed for this task and the spectra were recorded in the wavelength range from 190 nm to 700 nm with a resolution of 1 nm. The spectrophotometer was calibrated using methanol, which was also used for the dilution of the measured samples, so that their absorbance values would fit within the linearity limit of the method. Based on the calibration curve prepared earlier and absorbance values measured at 272 nm it was possible to determine the concentration of caffeine in the samples. The concentration values were averaged from three measurements and expressed as mole fractions.

### 3.5. FTIR-ATR Measurements

The FTIR-ATR analysis of solid caffeine precipitates was conducted based on the sediments collected from the test tubes obtained from the described above solubility experiments. After drying, the samples were measured with a FTIR Spectrum Two spectrophotometer from Perkin Elmer (Waltham, MA, USA) utilizing an ATR (attenuated total reflection) device. The wavenumber range was set to be 450–4000 cm^−1^.

### 3.6. Differential Scanning Calorimetry Measurements

The differential scanning calorimetry (DSC) measurements were also performed in order to characterize caffeine solid residues. The procedure involved co-grinding 0.4 g of caffeine samples with appropriate amounts of solvents. A Retsch (Haan, Germany) MM 200 mill was utilized for this task with a frequency of 25 Hz and 30 min duration. The specimens were ground using 5 mL stainless steel jars with two stainless steel balls and obtained powders were analyzed with a DSC 6000 from PerkinElmer (Waltham, MA, USA). The heating rate was set to 10 K/min and the inert atmosphere was provided by 20 mL/min nitrogen flow. The samples were placed in standard aluminum pans and before the measurements the apparatus was calibrated with the use of indium and zinc standards.

### 3.7. Affinities and Intermolecular Interactions

The solute-solvent affinities are characterized as a global measure by providing values of the Gibbs free energy of corresponding reactions, *ΔG_r_*, and also as decomposed sets of meaningful energetic contributions. The former value is computed as concentration independent quantity by assessments of activities in addition to the values of mole fraction equilibrium constant. For this purpose, COSMOtherm software [125] was invoked with the set of two default commands, namely “reaction” (for concentration-dependent values, *ΔG_r_^(x)^*) and “K_activity” (for concentration-independent values, *ΔG_r_^(a)^* [126]. As a result, for all considered solutions, the *ΔG_r_^(a)^* values are the same irrespectively of concentrations provided that the same qualitative composition is kept, whereas *ΔG_r_^(x)^* are allowed to be different for distinct binary compositions of the same ccNADES. Illustration of both types of affinities is provided in the Supplementary Materials (Figure S1). In this paper, the *ΔG_r_*^*(a)*^ quantity is used as a fundamental affinity measure. Apart from this, the computed values of interaction energies in the gas phase were augmented with the corrections accounting for zero-point vibrations, *ΔE^ZPE^*, electron correlation, *ΔE^corr^*, and basis superposition error, *ΔE^BSSE^*. It is important to emphasize that these contributions characterize weighted sums over all conformers included in the analysis. The weighting factors correspond to Boltzmann probability of a given conformer. Hence, the affinities were represented by the following reaction scheme: {*X_i_*} + {*Y*_*j*_} = {*XY_k_*} where *X*, *Y* stand for monomers and *XY* represents resulting pairs. In the case of *X* = *Y* a dimer is obtained, and otherwise hetero-molecular dimers are formed. In principle, each reagent is repressed by different conformers and in the above scheme *i*-th, *j*-th and *k*-th conformers are denoted in corresponding subscripts. The interaction energies account for weighted contribution of every conformer simply by the following sum:

(1) $$\Delta E_{XY}^{g,\omega }=\overset{N_{k}}{\underset{k=1}{\sum }}\omega_{k}\cdot \varepsilon_{k,XY}^{g,\omega }-\overset{N_{i}}{\underset{i=1}{\sum }}\omega_{i}\cdot \varepsilon_{i,X}^{g,\omega }-\overset{N_{j}}{\underset{j=1}{\sum }}\omega_{j}\cdot \varepsilon_{j,Y}^{g,\omega }$$

where *E* connotes the energy of the compound after averaging over individual energetic contributions, *ε*, of a given conformer.

The type of contributions is denoted by *ω* = {int, ZPE, corr}, and *N_i_*, *N_j_*, *N_k_* are the numbers of conformers of a particular species. Hence, this formula defines the way of computations of interactions energy (int), electron correlation (corr) and zero-point vibrational (ZPE) corrections. The necessity of extending the system characteristics beyond DFT was emphasized by Hellweg and Eckert [127]. The values of BSSE, *ω* = {BSSE}, were determined solely on the geometrical basis of the pair using reasonable simplification [128]. The final corrected intermolecular interactions energies in the gas phase can be computed by assuming the independence of all contributions in an additive way:

(2) $$\Delta E_{XY}^{g}=\Delta E_{XY}^{g,int}+\Delta E_{XY}^{g,corr}+\Delta E_{XY}^{g,ZPE}+\Delta E_{XY}^{g,BSSE}$$.

It is important to stress that each component of the above equation is computed on a different level of theory. The intermolecular interaction energies in the gas phase correspond to the results of full geometry optimization of both monomers and pairs using RI-DFT with BP97 GGA functional using def2-SVPD basis set and Grimme D3-BJ dispersion corrections [129]. The resulting geometries were used further for zero-point vibrational energy computations at the same level. Much more sophisticated computations were indispensable for accounting for the electron correlation. For this purpose, the single point energy computations were conducted at RI-MP2/def2-QZVPP level. Finally, BSSE was accounted on the same level as geometry optimization in the gas phase using gCP [128]. Since COSMOtherm is parametrized for BP86 (B88-VWN-P86) functional, it is necessary to generate “*cosmo*” and “*energy*” files which are coherent with BP_TZVPD_FINE_22.ctd parametrization. Hence, RI-DFT BP86/def2-TZVPD//BP86/def-TZVP computations were performed. This means that geometry was re-optimized using a smaller basis set, and single point energy was followed with a more extended one. These types of computations include the fine grid tetrahedron cavity and van der Waals dispersion term based on the “D3” method of Grimme et al. [129]. Completing this stage allows for computations of the values of Gibbs free energies of reactions involving solute and solvents molecules. The final values of affinity were computed as follows:

(3) $$\Delta G_{XY}^{\left(a\right)final}=\Delta G_{XY}^{\left(a\right)COSMO-RS}+\Delta E_{XY}^{g,corr}+\Delta E_{XY}^{g,ZPE}+\Delta E_{XY}^{g,BSSE}$$

All these contributions are collected in Table 1.

### 3.8. Solubility Computations

Theoretical solubility predictions were performed using the Conductor-like Screening Model for Real Solvents (COSMO-RS) approach. It relies on quantum chemistry computations applying the continuum solvation models based on which statistical thermodynamics analysis of electrostatic surface interactions is conducted for assessment of chemical potentials of components in the bulk phase. Although it is defined for liquid bulk systems, solids can also be considered if fusion thermodynamic characteristics is provided. There is no need for providing the details here, as they are available in original publications [130,131,132].

It is of crucial importance to mention, that the microscopic properties resulting from quantum chemical optimizations are used for generation of histograms of electron charge density, which are termed σ-profiles. Based on this information the thermodynamic macroscopic properties of components in the liquid systems are quantified by means of statistical thermodynamics through analyzing the densities probability distributions leading to so-called σ-potentials. This is sufficient for assessment of the combinatorial contribution to the chemical potential, leading eventually to such fundamental thermodynamic properties, as activity coefficients, excess properties, partition coefficients, phase diagrams, as well as solubility. Irrespectively of the computed property, a correct representation of the molecular structure is mandatory for adequate representation of statistics of segments surface electron densities. Hence, exploration of the conformational space is necessary. Here, the COMSOconf [133] software was used for generation of the most energetically favorable structures of all considered molecules. Taking advantage of BIOVIA TmoleX [134], computations were conducted using TURBOMOLE [135] software with RI-DFT BP86 (B88-VWN-P86) functional and def-TZVP basis set for geometry optimization and def2-TZVPD basis set for single point calculations with inclusion of the fine grid marching tetrahedron cavity. Additionally, the parameter sets with hydrogen bond interaction were included and van der Waals dispersion terms were quantified using the “D3” method of Grimme et al. [129]. All of the calculations of solubility were performed using COSMOtherm (version 22.0.0, revision 5273 M) [125] with BP_TZVPD_FINE_20.ctd parametrization.

Solubility computations of solid solutes were performed by iteratively solving the following equation:

(4) $$\log\left(x_{i}^{s,\left(k+1\right)}\right)=\frac{\mu_{i}^{o}-\mu_{i}^{s}\left(x_{i}^{s,\left(k\right)}\right)-\max\left(0,\Delta G_{fus}\right)}{RTln\left(10\right)}$$

where μ_*i*_^*o*^ stands for the chemical potential of a pure compound *i*, $\mu_{i}^{s}\left(x_{i}^{s,\left(k\right)}\right)$ is the solute chemical potential generated after *k*-th iteration with estimated solubility value equal to $x_{i}^{s,\left(k\right)}$ and ∆*G*_*fus*_ represents the solute Gibbs free energy of fusion. The starting point, for which *k* = 0, represents the zero-order approximation and characterizes solubility at infinite dilution conditions of the solute estimated using values of limiting activity coefficient, $x_{i}^{s,\left(0\right)}\approx 1/\gamma_{i}^{\infty }$. Equation (4) is solved iteratively until self-consistency is obtained by computing solubility using chemical potential estimated for mole fraction of the previous iteration. The form of solubility definition in Equation (4) requires the knowledge of the exact or at least approximated value of fusion Gibbs free energy, which must be provided from external measurements or estimations.

### 3.9. COSMO-RS DARE and Metafiles

Application of COSMO-RS for predicting physicochemical properties of any bulk system requires a proper representation of the chemical diversity of all components by defining a representative pool of conformations. This is not only related to the flexibility of the molecules adopting potentially many geometries but also to direct intermolecular interactions eventually leading to new compounds. This can originate from reactions occurring in the systems, for example, polymerization or hydrogen bonded complexes between solute solvent molecules. These species are outside of routine conformational analysis but might be important due to charge densities alterations affected by the very fact of complex formation. This problem is addressed by COSMO-RS with Dimerization, Aggregation and Reaction Extension (COSMO-RS-DARE) [96].

In this approach, the list of conformers of a given compound is extended by inclusion of products of any reaction, in which σ-surface includes only one molecule and weighting out all the rest of product. This ensures that the number of atoms and total formal charge suites monomeric forms not involved in the product formation. This is conducted via so-called “*mcos*” files, which have atomic weights set to one for atoms of considered species and zero to atoms of the second interacting component. Hence, such pseudo-conformers can be used for enriching the overall pool of conformers of a given compound. However, these interaction σ-surface segments of these pseudo-conformers are not captured with default settings of COSMO-RS and a new descriptor must be added ensuring that all new surface segments are included in computations. This is performed by introducing two adjustable parameters in the formal form of the interaction Gibbs free energy between *i*-th and *j*-th compounds:

(5) $$G_{ij}^{int}=H_{ij}^{int}-T\cdot S_{ij}^{int}$$

Hence, the COSMO-RS-DARE methodology computes interaction energy between surface segments including misfit, hydrogen bonding and van der Waals forces using the above formula of two interacting pseudo-conformers. The parameters of Equation (5) are estimated by fitting the computed solubility values to experimental ones for every solvent.

### 3.10. Contacts Conformational Analysis

The application of COSMO-RS DARE requires the definition of pseudo-conformers necessary for “*mcos*” files. Hence, conformational screening of bi-molecular systems is necessary, in which clusters of potentially probable structures are generated and optimized. The COSMOtherm program offers such kind of computation by performing the contact statistics based on the probability of interactions between molecule surface segments. Practically it is conducted by using “CONTACT = {1 2} ssc_probability ssc_weak ssc_ang = 15.0” command. This leads to automatic generation of series of geometries of high probability of contacts by alteration of the value of the dihedral angle between the two contacting molecules in the cluster with a step size of 15°. Weak interactions are also included in the probability statistics. The generated geometries were further optimized adopting the same scheme as for conformations analysis conducted for monomers. The number of pairs used for further analysis was restricted by the energy window of 2.5 kcal/mol, and pairs with stabilization energy above this threshold were discarded.

## 4. Conclusions

The solubility of caffeine was studied in several choline chloride-based natural deep eutectic solvents (ccNADES) and their mixtures with water. The results of performed optimization of the NADES component ratio suggested that unimolar proportion of cationic and anion counterparts had the highest ability of caffeine dissolution. The only exception was found for the system comprising choline chloride and glycerol in 1:2 molar ratio. Such ccNADES formulation was found to be the most effective among all seven polyolic components studied in 2:1, 1:1 and 1:2 molar ratios. Other ccNADES, particularly the ones utilizing sorbitol, xylitol and glucose, were also very effective in terms of caffeine dissolution. It was found that water and ccNADES exhibit co-solvency since the addition of a small amount of water to the ccNADES resulted in an increased caffeine solubility compared to the water-free ones. The composition involving a 0.8 molar ratio of ccNADES in the mixture turned out to be the most effective one. In general, it can be concluded that ccNADES perform better than standard organic solvents studied earlier, both in the water-free form and in aqueous mixtures. The usage of NADES is justified not only by their effectiveness but also by their beneficial health and environmental properties, especially when compared to classic organic solvents usually experiencing serious toxicity issues.

Such high solubilizing character ccNADES is granted from strong intermolecular interaction of caffeine with solvent constituents resulting in stable bi-molecular clusters formation. The highest contributions come from affinities of caffeine to sugars followed by interactions with choline chloride and water. Caffeine dimers are also allowed but these pairs are least stable among all considered intermolecular complexes. This is most probably related to stacking interactions rather than hydrogen bonding, although the latter is typical for all the rest of clusters. It was found that in all cases, the nitrogen center of the imidazole ring of caffeine plays the role of an accepter, while the hydroxyl group of the second counterpart of the hydrogen bonded complex acts as the hydrogen donor. It is interesting to note that not including these interactions in the default solubility computation performed with an aid of COSMO-RS approach results in caffeine solubility values which are at most in qualitative agreement with experimental data. This holds not only for saturated solutions of caffeine in neat solvents, but also in binary solvent mixtures and ccNADES. Fortunately, extending the system definition by adding interaction parameters dramatically increases accuracy leading to almost an exact match between measured and computed data. Unfortunately, the enthusiasm resulting from such effectiveness is tempered by the fact that fitting parameters cannot be obtained without prior experiments. However, the interaction parameters provide interesting characteristics of the studied systems and can be used for precise computation of solubility values for other formulations and temperatures. Moreover, fitting parameters used in COMSO-RS-DARE computations exhibit quite simple linear trends with ccNADES concentrations in aqueous mixtures. However, there is a clearly identifiable splitting of such linear trends around x_ccNADES_ ≈ 0.2. This observation was rationalized by diversities of intermolecular interactions between caffeine and water compared to the affinities to the rest of ccNADES constituents.

Finally, there has been an established correlation between caffeine fusion data and interaction parameters. This is a very interesting and valuable observation suggesting that COSMO-RS-DARE is able to compensate for errors of solubility data originating not only from inappropriate conformers definition in default COSMO-RS solubility computations but also inaccuracies or lack of thermodynamic data of fusion. This suggests that solubility computations can be extended also for systems for which the fusion data were not measured or are immeasurable. The latter case is quite common and originates from the fact that many organic solids are unstable in the melting temperatures, which results from decomposition below melting, high sublimation and solid forms transformations. Bearing in mind this feature of COSMO-RS-DARE, not reported so far, some important notes on a proper definition of “*meta*” files were provided. It was suggested that detailed analysis of σ-profiles similarities can effectively reduce the size of the optimization problem.

## Figures and Tables

**Table 1:** The values of intermolecular interactions (in kcal/mol) of caffeine in ccNADES systems.

Type of Interaction	$\Delta E_{XY}^{g,int}$	$\Delta E_{XY}^{g,corr}$	$\Delta E_{XY}^{g,ZPE}$	$\Delta E_{XY}^{g,BSSE}$	$\Delta E_{XY}^{g}$	Δ*G_r_*^(*a*)*COSMO-RS*^	Δ*G_r_*^(*a*)*final*^
C + C	20.20	0.22	25.57	0.18	31.66	0.12	38.27
C + ChCl	24.49	0.32	28.60	0.15	35.03	0.30	41.54
C + GL	28.41	0.46	33.52	0.28	39.94	0.33	47.57
C + SO	33.43	0.22	38.71	0.53	45.28	0.49	53.98
C + XY	39.27	0.25	46.85	0.60	54.10	0.63	64.45
C + GU	48.61	0.27	56.66	0.43	65.80	0.67	76.38
C + W	60.99	0.36	69.65	0.59	79.81	0.99	95.54

The following notation was used: caffeine (C), cholinę chloride (ChCl), glicerol (GL), sorbitol (SO), xylitol (XY), glucose (GU) and water (W). The last column comprises the final Gibbs free energy values of corresponding reaction in the bulk phase including all corrections (cor, ZPE and BSSE).

**Table 2:** The values of parameters defining linear relationship *G^int^* as a function of ccNADES composition derived from data presented in Figure 12.

$G^{int}=\left\{\begin{array}{c} \begin{array}{cc} \alpha \cdot 10^{3}\cdot x_{ccNADES}^{*}+\beta & x_{ccNADES}^{*}<0.2 \end{array}\\ \begin{array}{cc} \gamma \cdot 10^{3}\cdot x_{ccNADES}^{*}+\delta & x_{ccNADES}^{*}\geq 0.2 \end{array} \end{array}\right\}$		
System	α; β	γ; δ
[ChCl + glycerol + water]	21.93; −10.63	9.00; −5.68
[ChCl + sorbitol + water]	22.39; −10.99	8.84; −6.11
[ChCl + xylitol + water]	18.90; −9.93	4.35; −4.73
[ChCl + glucose + water]	27.73; −7.37	13.61; −12.62
[ChCl + glycerol + water]	21.93; −10.63	9.00; −5.68

**Table 3:** The values of fusion data characterizing solid caffeine.

Tm [K]	H_fus_ [kJ/mol]
509.05 ^1^ 510.2 [116], 510.3 [88], 508.8 [117], 508.7 [118], 505.4 [119], 510.0 [120], 507.7 [121], 509.6 [122], 509.1 [62], 510.8 [123]	20.66 ^1^ 20.08 [116], 19.52 [88], 20.37 [117], 19.60 [118], 17.90 [119], 20.80 [120], 24.80 [121], 23.46 [122], 20.29 [124], 19.44 [62], 20.95 [123]

^1^ mean value used in this work.

## Data Availability

Not applicable.

## Supplementary Materials

The following supporting information can be downloaded at: https://www.mdpi.com/article/10.3390/ijms23147832/s1.
