# Supplementary material for: Quantification of Caffeine Interactions in Choline Chloride Natural Deep Eutectic Solvents: Solubility Measurements and COSMO-RS-DARE Interpretation
Source: Int J Mol Sci. 2022 Jul 15;23(14):7832. doi: 10.3390/ijms23147832 (PMC9323268; doi:10.3390/ijms23147832)

Quantification of Caffeine Interactions in Choline Chloride Natural Deep Eutectic Solvents: Solubility Measurements and COSMO-RS-DARE Interpretation

Tomasz Jeliński[1,*] and Piotr Cysewski[1,*]

[1] Department of Physical Chemistry, Pharmacy Faculty, Collegium Medicum of Bydgoszcz, Nicolaus Copernicus University in Toruń, Kurpińskiego 5, 85-950 Bydgoszcz, Poland
* Correspondence: tomasz.jelinski@cm.umk.pl (T.J.); piotrc@cm.umk.pl (P.C.)

Supplementary materials

List of tables and figures

Table S1. Comparison of caffeine solubility values expressed as mole fractions ($\cdot 10^4$) obtained in this study and the results taken from literature. Standard deviation values ($\cdot 10^4$) are given in parentheses. Relative differences between datasets are also provided.
Table S2. Mole fractions ($\cdot 10^4$) and standard deviation (SD) values ($\cdot 10^4$) of caffeine in ccNADES at 25°C in different molar ratios (choline chloride first).
Table S3. Mole fractions ($\cdot 10^4$) and standard deviation (SD) values ($\cdot 10^4$) of caffeine in mixtures of water and ccNADES comprising choline chloride and glycerol in 1:2 molar ratio.
Table S4. Mole fractions ($\cdot 10^4$) and standard deviation (SD) values ($\cdot 10^4$) of caffeine in mixtures of water and ccNADES comprising choline chloride and sorbitol in 1:1 molar ratio.
Table S5. Mole fractions ($\cdot 10^4$) and standard deviation (SD) values ($\cdot 10^4$) of caffeine in mixtures of water and ccNADES comprising choline chloride and xylitol in 1:1 molar ratio.
Table S6. Mole fractions ($\cdot 10^4$) and standard deviation (SD) values ($\cdot 10^4$) of caffeine in mixtures of water and ccNADES comprising choline chloride and glucose in 1:1 molar ratio.
Scheme S1. Example of molecule definition for COSMO-RS DARE computations in caffeine + choline chloride + glycerol + water system.
Table S7. Graphical representation of "*cosmo*" and "*mcos*" files for caffeine pairs with constituents of choline chloride-glycerol-water system.
Table S8. The list of caffeine solubility in neat solvents taken from literature for presentation in Figure 11.
Table S9. The list of caffeine solubility in binary solvents taken from literature for presentation in Figure 11.
Table S10. Concentrations of caffeine solutions and the corresponding mean absorbance values with their standard deviations used during preparation of the calibration curve.
Table S11. Statistical parameters of the calibration curve used for caffeine solubility determination.
Figure S1. Illustration of concentration-related, $\Delta G_r^{(x)}$, and concentration independent, $\Delta G_r^{(a)}$, affinities in caffeine + choline chloride + glycerol + water systems. Plots correspond to the values of the Gibbs free energies estimated at room temperature (T=25°C) for caffeine dimer (AA) and caffeine pairs with choline chloride (AB1), glycerol (AB2) and (AB3) water, respectively.

Table S1. Comparison of caffeine solubility values expressed as mole fractions (·10^4) obtained in this study and the results taken from literature. Standard deviation values (·10^4) are given in parentheses. Relative differences between datasets are also provided.

dataset	15°C	25°C	30°C	35°C	40°C	45°C	55°C
**water**							
this study	-	20.19(±0.17)	25.56(±0.21)	31.35(±0.11)	38.26(±0.28)	-	-
Zhong 2017 [1]	12.30(±0.10)	16.10(±0.40)	-	22.70(±0.40)	-	29.60(±0.80)	39.80(±0.80)
		25.40%	-	*39.43%*			
Shalmashi 2010 [2]	-	20.98(±0.12)	26.21(±0.22)	30.75(±0.14)	43.70(±0.58)	-	-
		-3.77%	*-2.48%*	*2.93%*	*-12.38%*		
Dabir 2018 [3]	-	19.30(±0.60)	25.40(±0.80)	31.40(±0.90)	41.40(±1.20)	-	-
		4.61%	*0.63%*	*0.79%*	*-7.58%*		
**methanol**							
this study	-	20.75(±0.05)	26.35(±0.27)	33.01(±0.47)	41.65(±0.20)	-	-
Zhong 2017 [1]	13.40(±0.10)	18.90(±0.20)	-	28.00(±0.30)	-	42.60(±0.10)	66.30(±1.10)
		9.79%		*17.89%*			
Shalmashi 2010 [2]	-	20.04(±0.03)	26.38(±0.03)	34.09(±0.05)	41.94(±0.08)	-	-
		3.54%	*-0.12%*	*-3.17%*	*-0.70%*		
**ethanol**							
this study		16.04(±0.11)	19.52(±0.34)	22.69(±0.15)	26.65(±0.23)	-	-
Zhong 2017 [1]	7.80(±0.20)	13.20(±0.10)	-	20.40(±0.10)	-	32.20(±0.20)	46.90(±0.40)
		21.54%		*11.23%*			
Shalmashi 2010 [2]	-	17.13(±0.05)	20.46(±0.04)	21.88(±0.03)	26.45(±0.07)	-	-
		-6.35%	*-4.59%*	*3.71%*	*0.75%*		
**ethyl acetate**							
this study	-	38.30(±0.23)	43.27(±0.79)	49.67(±0.40)	57.67(±0.75)	-	-
Zhong 2017 [1]	27.00(±0.50)	35.40(±0.10)	-	48.80(±0.60)	-	68.90(±2.10)	92.40(±3.30)
		4.82%		*1.15%*			
Shalmashi 2010 [2]	-	40.46(±0.08)	44.59(±0.14)	48.36(±0.24)	55.22(±0.32)	-	-
		-5.34%	*-2.96%*	*2.72%*	*4.43%*		
**acetone**							
this study		42.03(±0.50)	54.10(±0.78)	65.56(±0.33)	78.00(±0.56)	-	-
Zhong 2017 [1]	27.90(±0.30)	36.30(±0.90)	-	52.80(±0.20)	-	72.80(±1.10)	95.30(±1.30)
		15.79%		*24.17%*			
Shalmashi 2010 [2]	-	45.35(±0.03)	57.59(±0.05)	69.77(±0.10)	84.70(±0.24)	-	-
		-7.32%	*-6.06%*	*-6.03%*	*-7.91%*		
**1-propanol**							
this study	-	18.55(±0.11)	22.85(±0.66)	28.52(±0.17)	35.81(±0.36)	-	-
Zhong 2017 [1]	11.70(±0.20)	17.70(±0.20)	-	28.20(±0.10)	-	45.30(±0.70)	71.60(±1.30)
		4.82%		*1.15%*			

[1] Zhong, J.; Tang, N.; Asadzadeh, B.; Yan, W. Measurement and Correlation of Solubility of Theobromine, Theophylline, and caffeine in Water and Organic Solvents at Various Temperatures. *J. Chem. Eng. Data* **2017**, *62*, 2570–2577. [2] Shalmashi, A.; Golmohammad, F. Solubility of caffeine in water, ethyl acetate, ethanol, carbon tetrachloride, methanol, chloroform, dichloromethane, and acetone between 298 and 323 K. *Lat. Am. Appl. Res.* **2010**, *40*, 283–285. [3] Dabir, T.O.; Gaikar, V.G.; Jayaraman, S.; Mukherjee, S. Thermodynamic modeling studies of aqueous solubility of caffeine, gallic acid and their cocrystal in the temperature range of 303 K–363 K. *Fluid Phase Equilib.* **2018**, *456*, 65–76.

Table S2. Mole fractions (·10^4) and standard deviation (SD) values (·10^4) of caffeine in ccNADES at 25°C in different molar ratios (choline chloride first).

NADES constituent	1:1 molar ratio		2:1 molar ratio		1:2 molar ratio	
	$x_C(\cdot10^{-4})$	SD($\cdot10^{-4}$)	$x_C(\cdot10^{-4})$	SD($\cdot10^{-4}$)	$x_C(\cdot10^{-4})$	SD($\cdot10^{-4}$)
glycerol	285.46	0.75	266.67	1.00	297.97	0.70
fructose	115.16	1.63	94.21	1.18	104.66	1.66
glucose	171.22	0.58	148.36	1.50	159.84	1.45
sorbitol	220.77	0.23	199.41	0.66	208.77	0.94
xylitol	207.36	0.82	180.55	0.44	187.84	0.34
sucrose	91.31	0.41	75.16	0.53	83.95	0.30
maltose	81.51	0.66	70.40	0.31	77.30	0.59

Table S3. Mole fractions ($\cdot10^4$) and standard deviation (SD) values ($\cdot10^4$) of caffeine in mixtures of water and ccNADES comprising choline chloride and glycerol in 1:2 molar ratio.

$x_{ccNADES}$	25°C		30°C		35°C		40°C	
	$x_C(\cdot10^{-4})$	SD($\cdot10^{-4}$)	$x_C(\cdot10^{-4})$	SD($\cdot10^{-4}$)	$x_C(\cdot10^{-4})$	SD($\cdot10^{-4}$)	$x_C(\cdot10^{-4})$	SD($\cdot10^{-4}$)
0.00	20.20	0.22	25.57	0.18	31.66	0.12	38.27	0.29
0.03	24.49	0.32	28.60	0.15	35.03	0.30	41.54	0.18
0.07	28.41	0.46	33.52	0.28	39.94	0.33	47.57	0.45
0.10	33.43	0.22	38.71	0.53	45.28	0.49	53.98	0.20
0.13	39.27	0.25	46.85	0.60	54.10	0.63	64.45	0.35
0.17	48.61	0.27	56.66	0.43	65.80	0.67	76.38	0.39
0.20	60.99	0.36	69.65	0.59	79.81	0.99	95.54	0.37
0.32	95.74	0.96	112.74	0.49	130.99	0.77	153.56	0.78
0.50	174.07	1.18	204.02	1.16	241.34	2.57	277.91	1.94
0.68	285.88	1.63	327.77	0.84	376.81	3.96	445.08	7.04
0.80	349.94	2.56	406.33	3.98	479.71	3.60	559.45	2.06
1.00	297.97	1.91	345.78	1.92	396.30	3.11	468.21	3.16

Table S4. Mole fractions (·10^4) and standard deviation (SD) values (·10^4) of caffeine in mixtures of water and ccNADES comprising choline chloride and sorbitol in 1:1 molar ratio.

$x_{ccNADES}$	25°C		30°C		35°C		40°C	
	x_C(·10^{-4})	SD(·10^{-4})	x_C(·10^{-4})	SD(·10^{-4})	x_C(·10^{-4})	SD(·10^{-4})	x_C(·10^{-4})	SD(·10^{-4})
0.00	20.20	0.22	25.57	0.18	31.66	0.12	38.27	0.29
0.03	23.46	0.24	28.02	0.26	33.98	0.29	41.04	0.08
0.07	26.42	0.32	30.80	0.28	37.81	0.17	47.10	0.38
0.10	29.93	0.15	35.75	0.31	43.21	0.24	51.07	0.23
0.13	36.07	0.41	41.95	0.22	47.97	0.29	57.72	0.21
0.17	42.76	0.48	49.76	0.42	58.81	0.31	68.29	0.35
0.20	49.29	0.43	57.98	0.46	67.78	0.36	78.97	0.38
0.32	80.35	0.62	92.41	0.82	108.18	0.60	128.20	0.87
0.50	149.67	1.35	172.50	0.67	198.44	1.06	232.99	1.23
0.68	222.12	2.32	255.35	1.17	303.30	1.35	359.93	1.18
0.80	255.84	2.10	299.92	0.10	353.07	1.47	413.65	2.09
1.00	220.77	0.66	252.55	1.66	298.22	2.01	351.96	2.08

Table S5. Mole fractions (·10^4) and standard deviation (SD) values (·10^4) of caffeine in mixtures of water and ccNADES comprising choline chloride and xylitol in 1:1 molar ratio.

$x_{ccNADES}$	25°C		30°C		35°C		40°C	
	x_C(·10^{-4})	SD(·10^{-4})	x_C(·10^{-4})	SD(·10^{-4})	x_C(·10^{-4})	SD(·10^{-4})	x_C(·10^{-4})	SD(·10^{-4})
0.00	20.20	0.22	25.57	0.18	31.66	0.12	38.27	0.29
0.03	22.03	0.34	27.02	0.23	32.95	0.19	40.78	0.16
0.07	25.21	0.38	30.74	0.26	36.69	0.28	45.24	0.23
0.10	29.47	0.28	34.53	0.27	41.61	0.22	50.10	0.34
0.13	34.16	0.46	40.00	0.32	46.91	0.20	55.98	0.35
0.17	41.01	0.22	49.44	0.37	57.13	0.21	66.74	0.45
0.20	48.45	0.20	56.50	0.37	66.13	0.25	77.07	0.13
0.32	77.01	0.53	89.88	0.80	105.72	0.62	123.72	0.26
0.50	142.50	0.47	165.82	1.18	192.70	1.16	223.46	1.16
0.68	212.65	0.94	246.33	0.89	287.24	0.95	340.23	0.88
0.80	248.68	1.49	288.47	2.79	337.20	1.69	394.23	2.43
1.00	207.36	1.20	242.28	1.92	283.81	3.34	335.15	0.63

Table S6. Mole fractions (·10[4]) and standard deviation (SD) values (·10[4]) of caffeine in mixtures of water and ccNADES comprising choline chloride and xylitol in 1:1 molar ratio.

$x_{ccNADES}$	25°C		30°C		35°C		40°C	
	$x_C(\cdot 10^{-4})$	$SD(\cdot 10^{-4})$	$x_C(\cdot 10^{-4})$	$SD(\cdot 10^{-4})$	$x_C(\cdot 10^{-4})$	$SD(\cdot 10^{-4})$	$x_C(\cdot 10^{-4})$	$SD(\cdot 10^{-4})$
0.00	20.20	0.22	25.57	0.18	31.66	0.12	38.27	0.29
0.03	21.34	0.21	26.40	0.21	32.41	0.26	40.21	0.19
0.07	23.82	0.13	29.60	0.17	35.59	0.15	44.07	0.45
0.10	26.48	0.22	32.81	0.26	39.37	0.10	48.11	0.26
0.13	31.12	0.18	36.67	0.26	44.68	0.23	53.12	0.16
0.17	39.09	0.35	46.14	0.29	54.16	0.36	63.81	0.24
0.20	47.50	0.39	54.73	0.57	64.84	0.34	76.30	0.24
0.32	74.42	0.52	86.55	1.30	102.21	0.73	119.28	0.70
0.50	130.09	0.77	153.80	0.23	184.85	2.04	217.66	1.14
0.68	196.04	1.82	228.12	2.19	267.78	1.40	312.31	1.11
0.80	226.08	4.34	261.93	0.78	302.32	2.77	356.94	1.17
1.00	171.22	0.77	196.33	0.41	232.41	1.79	274.96	2.76

Scheme S1. Example of molecule definition for COSMO-RS DARE computations in caffeine + choline chloride + glycerol + water system.

```
[f = A_c0.cosmo fdir = "directory" DGfus value Efile
 f = A_c1.cosmo fdir = " directory " Efile
 f = AB1.mcos fdir = "D:\BAZA_F2_kontakty_mcos\AB\ry\" IEI=1 Efile
 f = AB2.mcos fdir = "D:\BAZA_F2_kontakty_mcos\AB\ry\" IEI=1 Efile
 f = AB3.mcos fdir = "D:\BAZA_F2_kontakty_mcos\AB\ry\" IEI=1 Efile ]
f = ChCL fdir = "D:\BAZA_D2_BP\sg\" DGfus=0.0 autoc Efile
f = GL fdir = "D:\BAZA_D2_BP\pe\" DGfus=0.0 autoc Efile
f = W fdir = "D:\BAZA_D2_BP\xl\" DGfus=0.0 autoc Efile
```

The caffeine molecule (A) section is embraced by "[" and "]" brackets. First two lines includes two conformers of monomeric forms differing by rotation of methyl group. Next three lines define declaration of the "*mcos*" files with intermolecular contacts. The contended of these files is provided in the graphical way in Figure S7. The final three lines includes ccNADES constituents and water. The "autoc" stands for automatic inclusion of all conforms. Here, choline chloride is represented by 5 structures, glycerol by 10 conformers and water by one. The declaration of the values of adjustable parameter definition for COSMO-RS computations can be exemplified for 1:1 mixture as follows:

```
tc=25 solub xs={0 0 0 1} EN_IEI={1 1 -5.4080 0.0000}
```

Table S7. Graphical representation of "*cosmo*" and "*mcos*" files for caffeine pairs with constituents of choline chloride-glycerol-water system.

"*cosmo*" file of intermolecular complexes	"*mcos*" file with weightings identifying caffeine conformer within the pair
**caffeine dimer**	
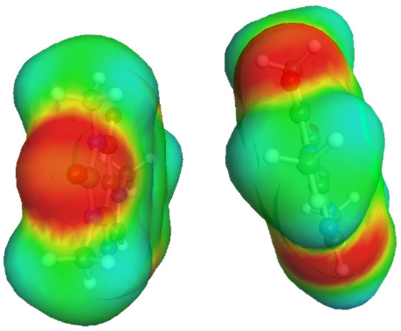	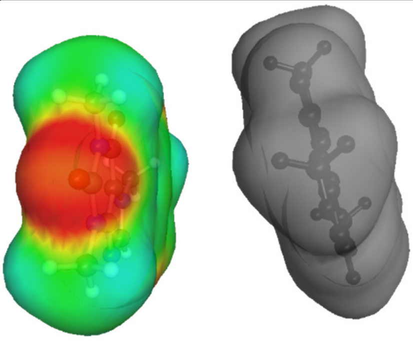 w={111111111111111111111111000000000000000000000000}
**caffeine-choline chloride**	
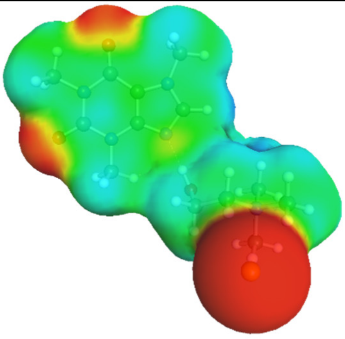	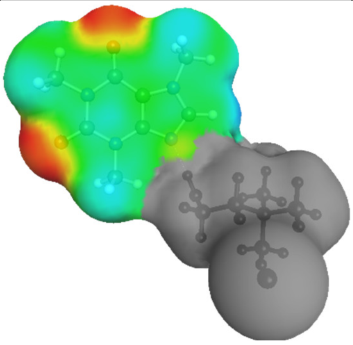 w={1111111111111111111111110000000000000000000000}
**caffeine-glycerol pair**	
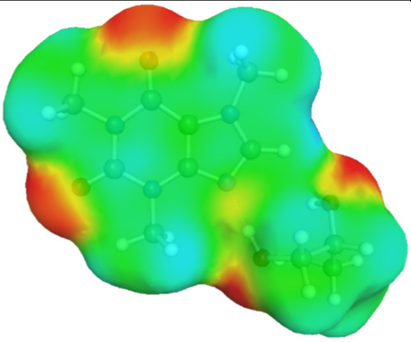	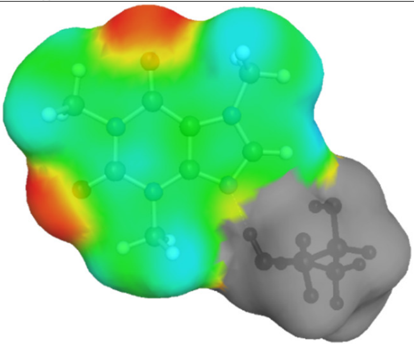 w={11111111111111111111111100000000000000}
**caffeine-water**	
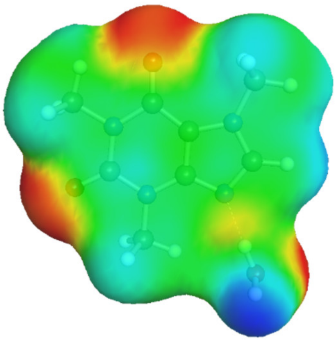	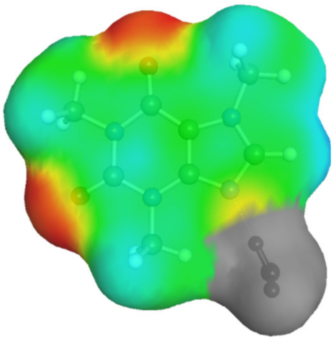 w={111111111111111111111111000}

Table S8. The list of caffeine solubility in neat solvents taken from literature for presentation in Figure 11.

solvent	datapoints	temperature range		literature
methanol	6	24.85	49.85	[1]
methanol	5	15.04	54.92	[2]
ethanol	5	24.85	39.85	[1]
ethanol	5	14.81	54.95	[2]
ethanol	10	20.05	40.05	[3]
1-propanol	5	14.86	55.00	[2]
1-propanol	5	20.05	40.05	[4]
isopropanol	5	20.05	40.05	[5]
ethylene glycol	5	20.05	40.05	[6]
propylene glycol	5	20.05	40.05	[7]
ethyl acetate	5	15.09	55.03	[2]
ethyl lactate	5	23.05	60.15	[8]
acetone	5	15.04	54.98	[2]
carbitol	5	20.05	40.05	[9]
carbon tetrachloride	12	24.85	49.85	[1]
chloroform	10	24.85	39.85	[1]
dichlormethane	5	24.85	44.85	[1]
N-methyl-2-pyrrolidone	5	20.05	40.05	[3.5]
water	5	14.86	55.00	[2]
water	6	24.85	49.85	[1]
water	10	24.85	69.85	[10]

[1] Shalmashi, A.; Golmohammad, F. Solubility of caffeine in water, ethyl acetate, ethanol, carbon tetrachloride, methanol, chloroform, dichloromethane, and acetone between 298 and 323 K. *Lat. Am. Appl. Res.* **2010**, *40*, 283–285.

[2] Zhong, J.; Tang, N.; Asadzadeh, B.; Yan, W. Measurement and Correlation of Solubility of Theobromine, Theophylline, and Caffeine in Water and Organic Solvents at Various Temperatures. *J. Chem. Eng. Data* **2017**, *62*, 2570–2577.

[3] Rezaei, H.; Rahimpour, E.; Ghafourian, T.; Martinez, F.; Barzegar-Jalali, M.; Jouyban, A. Solubility of caffeine in N-methyl-2-pyrrolidone + ethanol mixture at different temperatures. *J. Mol. Liq.* **2020**, *300*, 112354.

[4] Rezaei, H.; Jouyban, A.; Zhao, H.; Martinez, F.; Rahimpour, E. Solubility of caffeine in N-methyl-2-pyrrolidone + 1-propanol mixtures at different temperatures. *J. Mol. Liq.* **2021**, *346*, 117067.

[5] Rezaei, H.; Rahimpour, E.; Zhao, H.; Martinez, F.; Jouyban, A. Solubility measurement and thermodynamic modeling of caffeine in N-methyl-2-pyrrolidone + isopropanol mixtures at different temperatures. *J. Mol. Liq.* **2021**, *336*, 116519.

[6] Rezaei, H.; Rahimpour, E.; Martinez, F.; Zhao, H.; Jouyban, A. Study and mathematical modeling of caffeine solubility in N-methyl-2-pyrrolidone + ethylene glycol mixture at different temperatures. *J. Mol. Liq.* **2021**, *341*, 117350.

[7] Rezaei, H.; Rahimpour, E.; Zhao, H.; Martinez, F.; Jouyban, A. Determination and modeling of caffeine solubility in N-methyl-2-pyrrolidone + propylene glycol mixtures. *J. Mol. Liq.* **2021**, *343*, 117613.

[8] Manic, M.S.; Villanueva, D.; Fornari, T.; Queimada, A.J.; MacEdo, E.A.; Najdanovic-Visak, V. Solubility of high-value compounds in ethyl lactate: Measurements and modeling. *J. Chem. Thermodyn.* **2012**, *48*, 93–100.

[9] Rezaei, H.; Rahimpour, E.; Martinez, F.; Jouyban, A. Solubility of caffeine in carbitol + ethanol mixture at different temperatures. *J. Mol. Liq.* **2020**, *301*, 112465.

[10] Dabir, T.O.; Gaikar, V.G.; Jayaraman, S.; Mukherjee, S. Thermodynamic modeling studies of aqueous solubility of caffeine, gallic acid and their cocrystal in the temperature range of 303 K–363 K. *Fluid Phase Equilib.* **2018**, *456*, 65–76.

Table S9. The list of caffeine solubility in binary solvents taken from literature for presentation in Figure 11.

solvent 1	solvent 2	datapoints	temperature range		literature
N-methyl-2-pyrrolidone	ethanol	55	20.05	40.05	[1]
methanol	water	11	25.00		[2]
ethyl acetate	ethanol	55	5.00	40.00	[3]
carbitol	ethanol	55	20.05	40.05	[1]
N-methyl-2-pyrrolidone	isopropanol	55	20.05	40.05	[4]
N-methyl-2-pyrrolidone	1-propanol	55	20.05	40.05	[5]
N-methyl-2-pyrrolidone	ethylene glycol	55	20.05	40.05	[6]
N-methyl-2-pyrrolidone	propylene glycol	55	20.05	40.05	[7]
carbon tetrachloride	methanol	9	25.00		[8]
DMSO	water	48	25.00	40.00	[9]
DMF	water	48	25.00	40.00	[9]
1,4-dioxane	water	48	25.00	40.00	[9]
acetone	water	48	25.00	40.00	[9]

[1] Rezaei, H.; Rahimpour, E.; Martinez, F.; Jouyban, A. Solubility of caffeine in carbitol + ethanol mixture at different temperatures. *J. Mol. Liq.* **2020**, *301*, 112465.

[2] Cárdenas, Z.J.; Jiménez, D.M.; Almanza, O.A.; Jouyban, A.; Martínez, F.; Acree, W.E. Solubility and Preferential Solvation of Caffeine and Theophylline in {Methanol + Water} Mixtures at 298.15 K. *J. Solution Chem.* **2017**, *46*, 1605–1624.

[3] Bustamante, P.; Navarro, J.; Romero, S.; Escalera, B. Thermodynamic Origin of the Solubility Profile of Drugs Showing one or two Maxima Against the Polarity of Aqueous and Nonaqueous Mixtures: Niflumic Acid and Caffeine. *J. Pharm. Sci.* **2002**, *91*, 874–883.

[4] Rezaei, H.; Rahimpour, E.; Zhao, H.; Martinez, F.; Jouyban, A. Solubility measurement and thermodynamic modeling of caffeine in N-methyl-2-pyrrolidone + isopropanol mixtures at different temperatures. *J. Mol. Liq.* **2021**, *336*, 116519.

[5] Rezaei, H.; Jouyban, A.; Zhao, H.; Martinez, F.; Rahimpour, E. Solubility of caffeine in N-methyl-2-pyrrolidone + 1-propanol mixtures at different temperatures. *J. Mol. Liq.* **2021**, *346*, 117067.

[6] Rezaei, H.; Rahimpour, E.; Martinez, F.; Zhao, H.; Jouyban, A. Study and mathematical modeling of caffeine solubility in N-methyl-2-pyrrolidone + ethylene glycol mixture at different temperatures. *J. Mol. Liq.* **2021**, *341*, 117350.

[7] Rezaei, H.; Rahimpour, E.; Zhao, H.; Martinez, F.; Jouyban, A. Determination and modeling of caffeine solubility in N-methyl-2-pyrrolidone + propylene glycol mixtures. *J. Mol. Liq.* **2021**, *343*, 117613.

[8] Golubev, V.A.; Gurina, D.L. Dissolving power of the binary solvent carbon tetrachloride – methanol. Solubility of caffeine: Experiment, ASL model, and MD simulation. *J. Mol. Liq.* **2021**, *344*, 117736.

[9] Jeliński, T.; Kubsik, M.; Cysewski, P. Application of the Solute-Solvent Intermolecular Interactions as Indicator of Caffeine Solubility in Aqueous Binary Aprotic and Proton Acceptor Solvents: Measurements and Quantum Chemistry Computations. *Mater.* **2022**, *15*, 2472.

Table S10. Concentrations of caffeine solutions and the corresponding mean absorbance values with their standard deviations used during preparation of the calibration curve.

c [mg/ml]	A_{mean}	SD
0.0323	2.428	0.030
0.0258	1.942	0.038
0.0215	1.634	0.017
0.0185	1.398	0.020
0.0162	1.219	0.002
0.0144	1.074	0.018
0.0129	0.972	0.017
0.0118	0.896	0.023
0.0108	0.814	0.015
0.0099	0.733	0.015
0.0092	0.695	0.030
0.0086	0.648	0.023

Table S11. Statistical parameters of the calibration curve used for caffeine solubility determination.

parameter	value
a	75.338
b	$4.441 \cdot 10^{-16}$
R^2	0.999
LOD	$3.646 \cdot 10^{-4}$ mg/ml
LOQ	$1.094 \cdot 10^{-3}$ mg/ml

Figure S1. Illustration of concentration-related, $\Delta G_r^{(x)}$, and concentration independent, $\Delta G_r^{(a)}$, affinities in caffeine + choline chloride + glycerol + water systems. Plots correspond to the values of the Gibbs free energies estimated at room temperature (T=25°C) for caffeine dimer (AA) and caffeine pairs with choline chloride (AB1), glycerol (AB2) and (AB3) water, respectively.

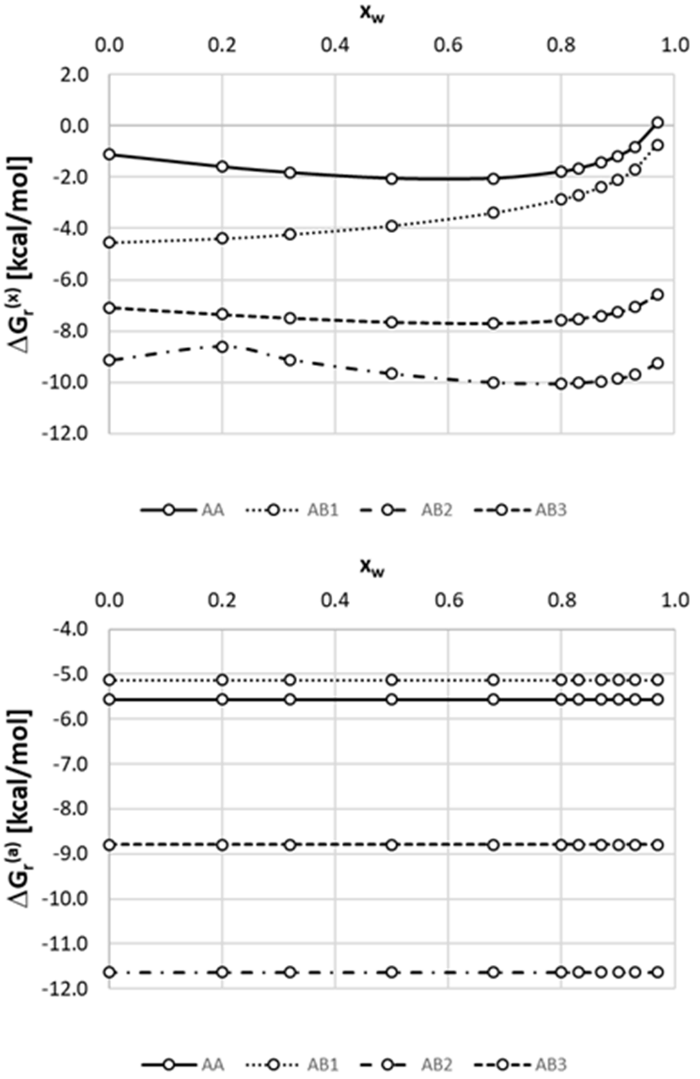

Supplement: Supplementary file 1 [file ijms-23-07832-s001.zip › ijms-1814364-supplementary.pdf]
